# Couple-based collaborative management model of type 2 diabetes mellitus for community-dwelling older adults in China: protocol for a hybrid type 1 randomized controlled trial

**DOI:** 10.1186/s12877-020-01528-5

**Published:** 2020-03-30

**Authors:** Jing Liao, Xueji Wu, Caixuan Wang, Xiaochun Xiao, Yiyuan Cai, Min Wu, Yuyang Liu, Xiongfei Chen, Shaolong Wu, Yung Jen Yang, Dong (Roman) Xu

**Affiliations:** 1 Department of Medical Statistics & Epidemiology, School of Public Health, Sun Yat-sen University, Guangzhou, P.R. China; 2 Sun Yat-sen Global Health Institute, School of Public Health and Institute of State Governance, Sun Yat-sen University, No.135 Xingang West Road, Guangzhou, 510275 P.R. China; 3 Division of Primary Health Care, Guangzhou Center for Disease Control and Prevention, Guangzhou, P.R. China; 4 School of Nursing, Sun Yat-sen University, Guangzhou, P.R. China; 5 Department of Health Policy and Management, School of Public Health, Sun Yat-sen University, Guangzhou, P.R. China; 6 Taiwanese Society of Geriatric Psychiatry, Taiwan, China

**Keywords:** Couple-based intervention, Social support, Type 2 diabetes, Self-management, Health behavior

## Abstract

**Background:**

China’s limited health care resources cannot meet the needs of chronic disease treatment and management of its rapid growing ageing population. The improvement and maintenance of patient’s self-management is essential to disease management. Given disease management mainly occurs in the context of family, this study proposes to validate a Couple-based Collaborative Management Model of chronic diseases that integrates health professionals and family supporters; such as to empower the couples with disease management knowledge and skills, and to improve the couples’ health and quality of life.

**Methods:**

The proposed study will validate a couple-based collaborative management model of Type 2 Diabetes Mellitus (T2DM) in a community-based multicenter, two-arm, randomized controlled trial of block design in Guangzhou, China. Specifically, 194 T2DM patients aged ≥55 and their partners recruited from community health care centers will be randomized at the patient level for each center at a 1:1 ratio into the couple-based intervention arm and the individual-based control arm. For the intervention arm, both the patients and their spouses will receive four-weekly structured group education & training sessions and 2 months of weekly tailored behavior change boosters; while these interventions will be only provided to the patients in the control group. Behavior change incentives will be targeted at the couples or only at the patient respectively. Treatment effects on patients’ hemoglobin, spouses’ quality of life, alongside couples’ behavior outcomes will be compared between arms. Study implementation will be evaluated considering its Reach, Effectiveness, Adoption, Implementation and Maintenance following the RE-AIM framework.

**Discussion:**

This study will generate a model of effective collaboration between community health professionals and patients’ family, which will shield light on chronic disease management strategy for the increasing ageing population.

**Trial registration:**

Chinese Clinical Trial Registry, ChiCTR1900027137, Registered 1st Nov. 2019

## Contributions to the literature

- Self-management is crucial to chronic disease management, but the adoption and adherence of self-management is generally poor among older patients.
- Our study explores how to incorporate older couples’ routine interactions into the disease management regime, and to build a collaborative management model with professional medical supervision and couple’s mutual support.
- This collaborative model provides innovative solutions for improving and maintaining older patients’ self-management behaviors; and facilitates the implementation of primary prevention for their informal carers mainly spouses at risk.

## Background

### Gaps in chronic disease management for older patients

China is ageing rapidly. In 2000, older people aged 65+ years were 7%, while the 2050 projection is 26%, reaching 365 million [1]. Due to this demographic shift, the number of older people living with chronic diseases is ever-increasing. The prevalence of chronic diseases among older people increased from 50% in 2003, 60% in 2008, to 72% in 2013; with a net growth of 22% over 10 years [2]. The healthcare system has been struggling to keep pace with the escalating chronic disease burden. The 2013 National Surveillance of Chronic Diseases and Risk Factors revealed that only 38% of older adults with diabetes received treatments and less than half of those treated were under control [3]. This treatment and management gap not only results from insufficient healthcare resources, but also is due to the lack of self-management awareness; leading to underutilization of community health services [4] and persistent unhealthy lifestyle [5]. Given the imbalance between the growing number of elderly patients and the shortage of qualified community health workers, how to improve and maintain self-management behavior of elderly patients is the key challenge of chronic disease management.

### Significance of spouse in chronic disease management

As self-management takes place in the context of families, family members, especially spouses, play a pivotal role in working with their loved ones in the management of chronic disease conditions [6, 7]. As such, the strained health workers shall leverage the daily interactions between the older couple, and incorporate the mutual efforts of the couple into the management plan. The proposed Couple-based Collaborative Management Model (CCMM) has the advantages of 1) facilitating mutual support between older couples in disease management, which has been associated with improved patient compliance [8] and quality of life [9]; 2) maintaining behavior change over long term as the couple’s shared unhealthy lifestyle can be addressed jointly [10]; and 3) preventing complications for the patients while prompting primary prevention for their spouses.

### Theoretical basis for CCMM

**Fig. 1:**
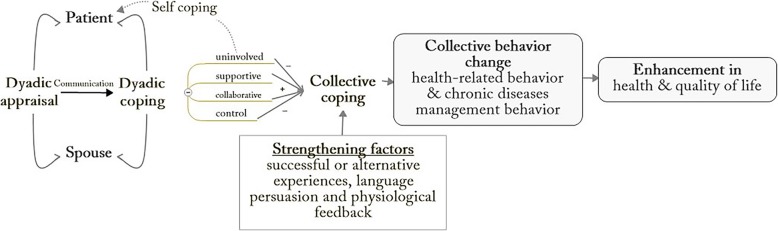
Theoretical framework for Couple-based Collaborative Management Model (+ Enhanced, − Reduced)

There are two main theories that best illustrate CCMM: the Dyadic Model of Coping with Chronic Illness (DMCCI) [11] and Social Cognitive Theory (SCT) [12] (Fig. 1). DMCCI proposed by Berg and Upchurch (2007) describes couples’ *dyadic appraisal* (e.g. illness severity, ownership, and management responsibility) and *dyadic coping* (i.e. uninvolved, supportive, collaborative or control) in chronic diseases management [11]. When patient and spouse both appraise the chronic disease as a shared problem that needs to be coped together, a *communal coping* is formed [13]. Building on *self-efficacy*, the core element of SCT [12], communal coping not only highlights the importance of family environment in patients’ behavior change; but also emphasizes the *collective efficacy* of couples in the dyadic coping process. Collective efficacy refers to the common belief held by the couple that they can cooperate to complete the management activities [14]. Efficacy can be strengthened by successful experiences (e.g. jointly developing and achieving goals), alternative experiences (e.g. acting as each other’s role models), language persuasion (e.g. mutual encouragement), and physiological feedback (e.g. emotional support). Strengthened collective efficacy can then trigger collective behavior change [13], which in turn is conducive to the couple’s health and overall quality of life.

### Previous research on CCMM

Research on CCMM has investigated several chronic diseases, like cancer, arthritis, cardiovascular disease or hypertension, chronic pain, type 2 diabetes mellitus (T2DM) [15] and depression [16]. Most of them are physical conditions, as mental diseases often lead to impaired cognition and communication barriers that make couple collaboration challenging to develop [11]. Physical health conditions that have long disease courses are susceptible to lifestyle and psychosocial interventions, and thus are suitable for CCMM [11, 17]. Intervention strategies employed by previous research fall into three broad categories [15]: couple-based health education, communication promotion, and behavior change training. These three strategies also have been implemented conjointly [15, 16] in the intervention group of no less than two pairs of couples, over 3 to 20 telephone or in-person courses, with comparison to a patient-only group with or without health education. The follow-up period ranges from 1 to 12 months.

So far, CCMM on chronic diseases management has been shown to improve patients’ depressive symptom [16], but its effects are not conclusive regarding couples’ behavior [10], patients’ physical condition [18], or spouses’ health status [6, 15]. There are few CCMM studies on T2DM. We found a total of five randomized controlled trials (RCT) on CCMM with diabetes. Although a positive ripple effect between couples on weight loss was found by Gorin and colleagues [19], the series of studies by Trief showed neither statistically-significant difference in blood glucose control among patients [20] nor significant changes in diet or physical activity levels of their spouses [21]. Another early RCT by Wing and colleagues suggested that the effect of CCMM may be gender-specific, such that female but not male diabetes patients were more like to lose weight if treated with their partners than treated alone [22]. To better evaluate the effects of CCMM, studies equipped with theory-driven interventions and outcome measures targeting at both the patients and their spouses are needed [6, 10, 15].

This proposed study is a type 1 hybrid implementation study in that we will primarily assess health outcomes in a real-world setting, while will also collect and examine implementation outcomes. We aim to evaluate the effects of CCMM in promoting health and wellbeing of Chinese older patients with T2DM and their partners living in communities; and explore implementation-related outcomes.

## Methods

### Study design

**Fig. 2:**
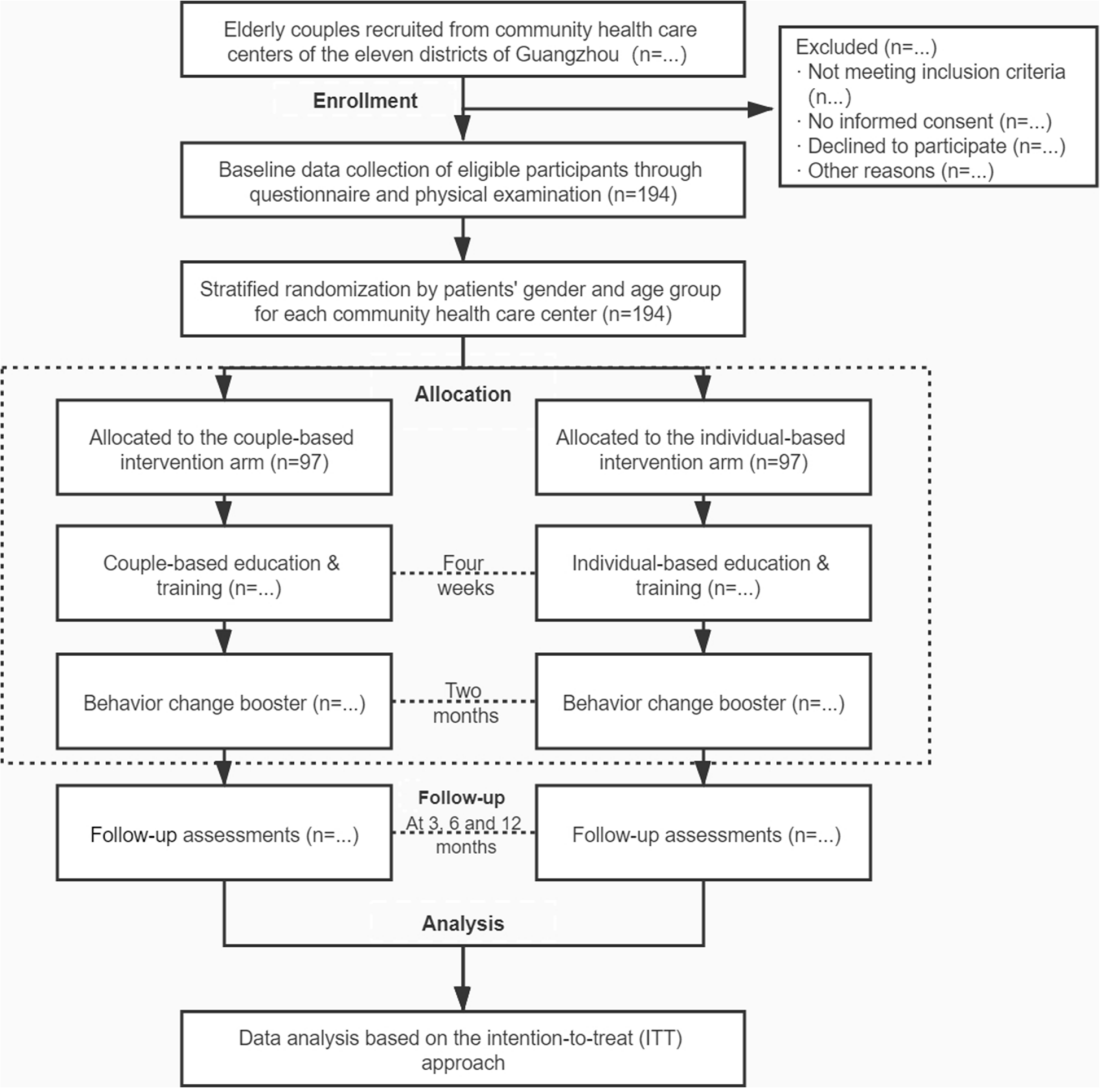
Study flowchart

The study is a community-based multicenter, two-arm RCT. To control for variations within community health centers, a randomized block design is adopted with couples recruited at each center (block) randomly assigned to the couple-based intervention arm or the individual-based control arm (Fig. 2). The study was approved by the Sun Yat-sen University Institutional Review Board (Approval no. 2019–064). The protocol was developed and guided by the SPIRIT checklist (Additional file 1).

### Setting

The study will be conducted in Guangzhou, Guangdong, China. Community health care centers of the 11 districts of Guangzhou that are willing to participate are selected as study implementation sites.

### Participants

Participants will be recruited from these selected sites. Eligible participants are older couples with one partner having poorly controlled T2DM, as defined below:

Eligible patients are these (1) confirmed T2DM patients who registered for the National Essential Public Health Services for T2DM Management; (2) with the latest fasting blood glucose level > 8.0 mmol/L or the latest glycosylated hemoglobin > 7.0% or newly diagnosed T2DM during the past 12 months; (3) aged ≥55 years; (4) with basic literacy, adequate cognitive and physical capability; (5) living with spouses; and (6) willing to provide informed consent to participate in the study. Patients who previously participated in T2DM education courses will be excluded.

Eligible spouses are these (1) married or cohabitate with a T2DM patient; (2) without mental or physical dysfunctions that may interfere with the study; and (3) willing to provide informed consent. We further exclude couples that both have diabetes, for a clear distinction between patients and spouses.

### Recruitment

For participant recruitment, community health care centers will first identify all potentially eligible participants from their case management system. Community nurses will contact these patients and their spouses to inform them about the study by phone or in person when patients visit the centers. In addition, awareness campaigns and publicity will be conducted at the centers, through onsite posters, publicity materials and advertisements broadcasted on the centers’ website and WeChat accounts.

### Sample size

The sample size calculation was based on the primary hypothesis that the couple-based intervention would result in a 0.5% more reduction in HbA_1c_ as compared to the individual-based intervention over 12 months. This difference in HbA_1c_ is interpreted as a clinically meaningful change in glycemic control [23, 24]. A sample size of 194 couples (97 couples per arm) would provide 80% power to detect a between-arm difference of this magnitude (standard deviation (SD) =1.5%) for a longitudinal design with three repeated measures having an autoregressive covariance pattern, assuming the correlation between observations on the same subject of 0.5 [25], *α* of 0.05, and a 10% dropout rate.

The proposed sample size also satisfies the power requirement for subgroup analyses among male patients and patients with baseline HbA_1c_ ≥ 8.0%. These two subgroup analyses were chosen, considering the traditional female-dominated role in meal preparation and caregiving [26]; and a more evident reduction in HbA_1c_ among those with worse baseline glycemic control [20]. In light of previous couple-based T2DM interventions [20, 27, 28], it is assumed the ratio of male vs female patients enrolled would be around 1.2, resulting in 106 male participants available for subgroup analysis. With *α* of 0.05 and an auto-correlation of 0.5, the study will have over 90% power to detect a between-arm difference of 0.6% in HbA_1c_ (SD = 1.1%). Similarly, for the subgroup of patients with HbA_1c_ ≥ 8.0% (assuming *n* = 64, 33% of the whole sample), our study will have 90% power to detect a between-arm difference of 0.7% in HbA_1c_ (SD = 1.1%). Sample size and power calculation were conducted by PASS 11, using two independent means with repeated measures procedure [29] .

### Randomization and allocation concealment

Participants of each community health center will be randomized 1:1 within center into a couple-based arm (intervention) and an individual-based arm (control). To better balance key covariates, stratified randomization by patients’ gender and age group will be performed by a statistician otherwise not involved in the project using STATA 15. The order of which arm receiving each education session first will also be randomized, to ensure that the treatment sequence is equivalent between arms. Other than the statistician, all the research staff and community health workers will not know the group allocation prior to the education session. The community health workers designated as the care managers will call the participants to inform them of their group allocation, and to schedule the first session.

### Procedures

As outlined in Table 1, the intervention and control arms will take four-weekly group education & training sessions, and receive behavior change booster calls over the following 2 months. The education content will mainly be based on the T2DM management program [31] covering the topics of diabetes and complications, healthy diet, medication and exercise; and embody behavior change techniques (BCTs) [32]. During the intervention, patients will maintain their routine treatment. Interventions are targeted at both the patients and their spouses for the intervention arm, while only at the patients for the control arm.

**Table 1:** Intervention components for intervention and control arms

Module	Dosage & Delivery	Components	Couple-based Intervention Group	Individual-based Control Group	BCT ^**a**^
**Health Education & Training**	4 weekly 2-h sessions, delivered by two care managers in class.	**Group Education**	T2DM patients & their spouses	T2DM patients	Comparison of behavior
		**1. Diabetes** & **Complication**			
		Understand diabetes Hypoglycemia Glucose monitoring	Couple-level discussion & practice	Patient-level discussion & practice	Shaping knowledge
		**2. Healthy Diet**			
		Diabetes nutrition Food label Dietary plan	Collaborative- management Collective behavior goal setting	Self-management Individual behavior goal setting	Social support ^b^ Goals & planning
		**3. Medication**			
		Taking (multi-) medication Medication adherence Foot care	Couples identify barriers & solutions collaboratively;	Patients identify barriers & solutions themselves;	Antecedents Association
		**4. Being Active**			
		Exercise recommendations Risk management Exercise goal & plan	Use “we will” in these activities.	Use “I will” in these activities.	Goals & planning
**Behavior Change Booster**	2 months of weekly tailored call	Interactive call tailored to participant’s behavior change barriers, with call frequency varied by their progress.	Deliver to the couple	Deliver to the patient only	Repetition & substitution Feedback & monitoring
**Behavior Change Incentive**	Throughout intervention	Vouchers gain or lose by fulfilling or failing management tasks, with group ranking.	Incentives for individual & couple performance	Incentives for individual performance only	Reward & threat

^a^ BCT: behavior change technique was defined by Behavior Change Techniques Taxonomy Volume 1 [30]

^b^ BCT for couple-based intervention only

### Interventions

#### The individual-based education & training

The four weekly two-hour structured group-education sessions focusing on T2DM management will be attended by eight to ten patients and facilitated by two care managers. The patients’ spouses, other family members or friends will not participate in these sessions. Discussion and practice will be organized to reflect patients’ own health and daily management issues. At the end of each session, patients will set a health-related behavior goal (e.g. exercise three times over the next week) and will report their progress in the next session with the group. Primary BCTs utilizes are social comparison, sharping knowledge, natural consequences, and goals and planning.

#### The couple-based education & training

The couple-based education groups consist of eight to ten couples (patients and their spouses) and two care managers. The education and training components will be framed as the couple’s issues whenever possible, and couple-level discussions and practices are interspersed throughout the sessions. The couples are encouraged to share their pragmatic aspects of coping with T2DM, and care managers will provide knowledge and techniques on how spouses can aid the management process accordingly.

At the end of each session, couples will set health-related behavior goals and make plans for the following week, which will be reviewed among the group at the beginning of the next session. They will be instructed to make these plans following the collaborative implementation intentions [30], that is the joint self-regulatory strategies involving both partners in planning when and where to perform a specific behavior task. Couples will set their personalized behavior goals, and make plans regarding when, where and how to achieve those goals collaboratively. Specifically, they are encouraged to list potential barriers in achieving these goals and identifying the best ways for the partner to help each other overcome these barriers. Spouses are instructed to set goals facilitative to their partners’ goals whenever possible (Additional file 2. Example for physical activity plan & goal).

The care managers serve as a resource to facilitate discussion within and between couples, and to instruct patients and their spouses to collaboratively make behavior plan. They are also trained to be aware of the spouses’ health condition, who are likely to have multiple chronic conditions themselves. In addition to the BCTs utilized in the individual-based education & training, couple-based education sessions further demonstrates restructuring the physical and social environment as antecedents of behavior change, as well as practical and emotional support from the closest person.

#### Behavior change booster

To reinforce and monitor participants’ progress in T2DM management, weekly booster calls will be delivered over the following 2 months. Given personalized messages with tailored feedback and frequency are more effective in promoting behavior changes [33], booster calls are designed to address participant’s behavior change barriers and call frequency are varied by participants’ progress. During the first call, trained investigators will examine the extent to which the patients have fulfilled behavior change action plans corresponding to the four education sessions, and provide motivational, informative and problem-solving feedbacks accordingly. Follow-up calls will be scheduled based on patients’ performance and only targeted at these behaviors that needs further attention. For example, if the patients fulfilled two out of the four behavior plans, the following week’s call will only address the other two unsolved behavior change tasks. If the patients have achieved all behavior change goals, they will only be contacted after another 3 weeks to review maintenance. While only the patients of the control arm will be contacted, both the patients and their spouses of the intervention arm will receive the call. Spouses are encouraged to review the couples’ progress jointly (e.g. healthy diet and exercise); and discuss ways to help the patients achieve the behavior change goals (e.g. glucose monitoring) and will receive suggestions from the investigators. BCTs associated with this module are repetition and substitution, as well as feedback and monitoring.

#### Behavior change incentives

To assist the delivery of the intervention, we further incorporate the reward and threat BCTs addressing both material and social incentives and rewards. Considering evidence from behavioral economics, incentives are designed to leverage the persons’ loss aversion [34] and social influences [35]. Participants of both arms will be given same amount of virtual vouchers upon enrollment. They can win extra vouchers by attending education sessions and fulfilling management tasks; or lose these vouchers vice versa. In the individual-based control arm, gain or loss depends on the patient own behavior only. In contrast, for the couple-based intervention arm, participants’ gain or loss not only depends on their own behavior, but also on the collaboration of the couples, meaning that the award or penalty will be doubled if both of them complete or fail the task. The total number of vouchers will be ranked and announced within their education group; which can be used to redeem gifts at the end of the course.

#### Intervention fidelity

The interventions of both arms will be delivered by two community health workers of each selected health care center. These designated care managers will receive ten-hour training of the interventions prior to the first group education session, and will be supervised by the research team while implementing the interventions. To maintain the fidelity of the intervention delivery, care managers will follow the manualized interventions, and take a structured fieldnote questionnaire (e.g. length of each session, any interruptions, participant dynamic and mood etc.). The educational sessions will be taped and monitored. In addition, participants’ attendance rates for each session and reasons for non-attendance will be collected for the implementation evaluation.

#### Study measures

Patients and their spouses will complete the baseline questionnaire and physical examination. Follow-up assessments are at 3 (immediately after intervention), 6, and 12 months after baseline (Table 2). Research assistances will conduct the two assessments on behavioral and management efficacy for both arms at 3 and 6 months via telephone. Patients of both arms will maintain their routine doctor appointments scheduled at the 6-month follow-up when their venous blood sample will be collected. Following the standard requirements for blood transportation, the blood sample will be mailed to Daan Gene, a university-affiliated clinical laboratory for examination. The endpoint in-person assessment at the 12-month will be conducted at community health care centers for all participants. All data obtained will be double entered into and managed by the Research Electronic Data Capture (REDCap) system [36, 37] hosted at hosted at Sun Yat-sen Global Health Institute. Outcome assessors will be blinded to the group assignments and will be different from care managers who conduct and monitor the interventions. Data analysts will remain blinded to the group assignment throughout the study.

**Table 2:** Schedule of enrolment, interventions, and assessments

		STUDY PERIOD					
	Enrolment	Baseline Assessment	Allocation	Intervention	Follow-up		
**TIMEPOINT** (T for Month)	**T**_**−2**_	**T**_**−1**_	**T**_**0**_	**T**_**1–3**_	At end of **T**_**3**_	At end of **T**_**6**_	At end of **T**_**12**_
**ENROLMENT:**							
Eligibility screen	X						
Informed consent	X						
**ALLOCATION**			X				
**INTERVENTION:**							
Couple-based Intervention Group				X			
Individual-based Control Group				X			
**ASSESSMENTS AND MEASUREMENT:**							
Baseline socio-demographics: **age, gender, marital status, education, retirement status**, etc.	X						
Mental health: **C-MMSE**, **CES-D**		X					
***Primary outcomes:***							
Blood glucose ^a^: **HbA**_**1c**_		X				X	X
Quality of life: **SF-36**		X				X	X
***Secondary outcomes:***							
Metabolic measures: **BP, BMI, WHR, FG & lipid**		X					X
Management behaviours ^a^: **SDSCA**		X			X	X	X
Medication adherence ^a^: **BMQ & medical records**		X			X	X	X
Physical activity ^b^: **IPAQ-C**		X			X	X	X
Dietary ^b:^**FFQ**		X				X	X
***Process measures:***							
Management efficacy ^b^: **C-DMSES**		X			X	X	X
Dyadic appraisal & coping ^b^: **Communal coping** & s**upport**		X			X	X	X

*C-MMSE* Chinese Mini-Mental State Examination, *CES-D* the Centre for Epidemiologic Studies Depression Scale, *HbA1c* hemoglobin A_1c_, *SF-36* 36-item short form survey, *BP* blood pressure, *BMI* body mass index, *WHR* waist hip ratio, *FG* fasting glucose, *SDSCA* summary of diabetes self-care activities, *BMQ* brief medication questionnaire, *IPAQ-C* International Physical Activity Questionnaire-Chinese, *FFQ* food frequency questionnaire, *C-DMSES* the Chinese version of the Diabetes Management Self-efficacy Scale

^a^ Measure for patients only

^b^ Repeat measure for spouses of the control arm only at baseline and 12 months

#### Primary outcomes

The primary outcome for the patients is glycemic control, as measured by mean changes in HbA_1c_ levels over the 12-month follow-up. HbA_1c_ is an indicator of average blood glucose over the past 2 to 3 months [24].

For the spouses, health-related quality of life as measured by the 36-item Short Form Survey (SF-36) [38] is selected as the primary outcome. SF-36 covers physical functional health and well-being scores and has been validated among Chinese older adults [39]. Patients will also take the SF-36 survey as one of their secondary outcomes.

#### Secondary outcomes

Metabolic outcomes for both the patients and their spouses include waist/hip ratio, body mass index (BMI), blood pressure, fasting glucose and lipid profile.

Patients’ diabetes management behaviors will be assessed by the Summary of Diabetes Self-Care Activities (SDSCA) questionnaire [40]. SDSCA has 12 items evaluating the number of days in the past week that patients perform exercise, healthy diet, glucose monitoring, foot care, and medication adherence. The more days they conduct these self-care activities as required, the higher the score will be. The SDSCA has been validated among Chinese T2DM patients [41]. Medical adherence will be assessed by medical record and the brief medical questionnaire [42], screening patient adherence and barriers to adherence.

Physical activity behavior will be measured by the International Physical Activity Questionnaire (IPAQ)-Short Form [43], which evaluates the frequency and duration of four types of physical activities over the past week. The validity and reliability of IPAQ-Chinese has been previously established among older Mainland Chinese [44]. Dietary will be evaluated by the food frequency questionnaire customized to the Chinese Chronic Non-Communicable Diseases and Risk Factor Surveillance questionnaire [45, 46], to assess food intake and changes in eating behaviors over the past 6 months. These two behavior measures will be applied to patients and spouses.

#### Process measures

Management efficacy will be examined by the Chinese version of the Diabetes Management Self-efficacy Scale (C-DMSES) [47]. The 20-item C-DMSES assesses patients’ confidence in conducting diabetes management daily activities from 0 (can’t do at all) to 10 (certain can do). Spouses will also evaluate their confidence in assisting the patients completing these activities on the same 11-point scale [48].

Couples’ dyadic appraisal and dyadic coping behaviors of diabetes will be examined by questionnaires synthesized by Helgeson et al., 2019 [49]. Patients and their spouses will individually evaluate whether they think diabetes is an individual or shared problem (dyadic appraisal) [50], and the extent to which they had engaged in collaborative supportive and unsupportive activities during the past month on a five-point Likert scale from 1 (not at all) to 5 (very often) (dyadic coping).

#### Statistical considerations

Descriptive analyses comparing couples’ baseline characteristics between the intervention and control arms will be performed to assess the balancing of the group assignment. The intention-to-treat (ITT) approach will be applied to examine the treatment effects in accordance with the original randomization assignments. Missing baseline covariates and missing outcomes will be multiply imputed under missing at random assumption and separately by treatment arms [51].

The multilevel linear mixed modeling (MLM) will be employed to evaluate repeat measures of participants over time, while accounting for variations between community health care centers (blocks). The longitudinal changes of patients’ HbA_1c_ within- and between- treatment arms over 12 months will be examined, and the treatment effect is estimated as the differences between the within-arm changes from baseline to follow-up between the two intervention arms. MLM will be also applied to other continuous outcomes with one or more repeat measures, adjusted for time, study arm, a time and study arm interaction, and baseline covariates. The treatment effect will be further examined in two subgroup analyses, stratified by gender and patients’ baseline glycemic levels. All analyses will adjust for baseline sociodemographic covariates and functional states that are statistically-significantly different between treatment arms, and empirically-suggested as strong predictors for the outcomes.

#### Implementation evaluation

The study implementation will be evaluated in reference to the Reach, Effectiveness, Adoption, Implementation, and Maintenance (RE-AIM) framework [52, 53]. Tailored to the present study (Table 3), Reach will be revaluated by participation rate, and representativeness of participants. Effectiveness will be indicated by the health outcomes listed in Table 2 above; and Maintenance at the participants level will be measured as these treatment effect for long term (i.e. 1 year).

**Table 3:** Reach, Effectiveness, Adoption and Implementation (RE-AIM) framework indicators

Domain	Indicators	Source	Data Collection Tool
**Reach**	Participation rate	Participants & Disease management system	Enrolled couples /all couples being contacted
	Representativeness		Enrolled couples / all eligible couples registered in the system
**Effectiveness**	Health outcomes	Participants	HbA_1c,_ Quality of life, BMI, WC, blood pressure, fasting glucose & lipid profile; management and healthy behaviors (exercise, diet)
**Adoption**	Number of health workers who prefer couple-based over individual-based interventions	Community health workers	Qualitative interview and survey of community health workers
**Implementation**	Treatment fidelity	Randomly selected taped sessions	10% of taped sessions randomly selected and reviewed by an expert panel, against the full detailed intervention manuals for adherence and quality.
	Participant involvement	Participants	Course registration forms recording attendance rate
	Participant satisfaction	Participants	Satisfaction questionnaire on the program, from 1 (strongly disagree) to 4 (strongly agree).
	Incremental cost- effectiveness ratio (ICER) of the intervention and control arms	Participants	ICER = ∆C/∆E = (C_intervention_ − C_control_)/ (E_intervention_ – E_control_); C is the program and labor costs; E is effectiveness defined as the percentage of patients whose HbA_1c_ is lower than 7%
**Maintenance**	Effectiveness over 1 year	Participants	Same as effectiveness.

Adoption by the community health workers will be calculated as the number of them who prefer the couple-based intervention than the individual-based intervention over total number of care providers conducting the interventions.

Implementation will be examined by treatment fidelity, participant involvement and satisfaction with the program, and cost-benefit. Treatment fidelity will be evaluated by an independent expert of behavior medicine, who will randomly select 10% of the taped sessions and evaluate them against the fully detailed intervention manuals for adherence (whether contents are being presented as intended) and quality (how well sessions are being delivered). Participant involvement will be measured by the attendance rate. Participants’ satisfaction with intervention will be evaluated by the degree to which they agree with a series of statements, on a scale from 1 (strongly disagree) to 4 (strongly agree) (Additional file 3). The cost-benefit will be measured as the incremental cost-effectiveness ratio (ICER) between the intervention and control arms. The ICER is calculated as the difference in program and labor costs divided by the difference in effectiveness of the two treatment arms, whereby effectiveness is defined as the percentage of patients reached HbA_1c_ < 7%.

#### Trial status

The study recruitment will start by the end of March-2020.

## Discussion

There is increasing demands for chronic disease management among community-dwelling older adults. The proposed study aims to empower older couples through constructing a collaborative management model supervised by medical professionals. It targets the older patients, and their closest persons who not only are the main resources for informal caregiving, but also are likely to be burdened with chronic diseases themselves. This study may further serve the purpose of optimizing the strained community health resources.

### Strengths & limitations

The development of the couple-based collaborative management model is based on theoretical framework, the latest clinical guidelines and the needs of old couples and community health workers. The model validation uses a randomized controlled trial design under strict quality control and makes thorough effect evaluation regarding its effects on the patients, their spouses and the whole implementation process. Our study will be conducted at the community health care centers that have established stable doctor-patient relationships with the elderly dwellers. This solid patient foundation will facilitate our participant recruitment and follow up.

The main limitation of our study is its external validity. Older couples that are willing to participate together may have better marital relationships and are open to behavior changes. This participants’ selection bias may not affect the study’s internal validity thanks to the randomized controlled study design, whereas its implication to broader older couples may need further examination.

### Significance

Given the ever-growing elderly population and cumulating empty-nest families in China, the proposed study explores how to incorporate older couples’ routine interactions into the chronic disease management regime, and to build a collaborative management model with professional medical supervision and couple’s mutual support. Our study will generate empirical evidence for maximizing the use of limited community healthcare resource and personnel, through leveraging family support. Moreover, by empowering couples with disease management knowledge and skill, our study provides innovative solutions for patients’ behavior change and maintenance, as well as primary prevention for their spouses at high risk. Once proved effective, this interdisciplinary study has the potential to improve the health and quality of life of millions of older diabetes patients and their family.

## Supplementary information

**Additional file 1.** The SPIRIT reporting guidelines. The SPIRIT 2013 checklist our manuscript follows according to the recommendations on it.

**Additional file 2.** Activity planning demonstration. A demonstration of the intervention implemented on participants specific to each single day of the week.

**Additional file 3.** Evaluation of couple intervention. Evalutaion of patients and their spouses during intervention implementation.

## Data Availability

Data collected during the study will be securely stored and managed by the Research. electronic data capture (REDCap) system in China. All study records and documents will be anonymized and stored for 10 years from the end of the study. Informed consent will be obtained from all individuals contributing data by the research assistances. Study findings will be published on peer reviewed journals, and public access to the study raw data will be available upon approval via the Chinese clinical trial registry.

## Abbreviations

BCT: Behavior change techniques
BMI: Body mass index
CCMM: Couple-based collaborative management model
C-DMSES: Chinese version of the Diabetes Management Self-efficacy Scale
C-MMSE: Chinese Mini-mental State Examination
CES-D: the Centre for Epidemiologic Studies Depression Scale
DBP: Diastolic blood pressure
DMCCI: Dyadic model of coping with chronic illness
FPG: Fasting plasma glucose
HbA1c: Glycated hemoglobin
HDL-C: High density lipoprotein-cholesterol
ICER: The incremental cost- effectiveness ratio
IPAQ: The International Physical Activity Questionnaire
ITT: The Intention-to-treat
LDL-C: Low density lipoprotein-cholesterol
MLM: The multilevel linear mixed modeling
NPT: Normalization process theory
RCT: Randomized controlled trial
RE-AIM: The reach, effectiveness, adoption, implementation, and maintenance
SBP: Systolic blood pressure
SCT: Social cognitive theory
SD: Standard deviation
SDSCA: The summary of diabetes self-care activities
FFQ: Food frequency questionnaire
SF-36: The 36-item short form survey
T2DM: Type 2 diabetes mellitus.

## References

[1] United Nations (2019). World Population Prospects 2019.

[2] Caiyou W, Qun M, Ling X, Yude C (2015). An analysis report of national health services survey in China (2013).

[3] Wang L, Gao P, Zhang M, Huang Z, Zhang D, Deng Q (2017). Prevalence and ethnic pattern of diabetes and Prediabetes in China in 2013. JAMA..

[4] Xiaoli Z (2017). Current problems and thinking in the management of chronic diseases in the elderly in our community. Community Med J.

[5] Ping T, Jianqun D (2011). Progress in self-management of diabetic patients. Prev Control Chronic Dis China.

[6] Martire LM, Helgeson VS (2017). Close relationships and the management of chronic illness: associations and interventions. Am Psychol.

[7] Toukhsati SR, Hare LD (2016). Towards optimal heart failure care: couples-oriented strategies to improve patient adherence and health outcomes. Curr Cardiol Rev.

[8] Dimatteo MR (2004). Social support and patient adherence to medical treatment: a meta-analysis. Health Psychol.

[9] Liao J, Brunner EJ (2016). Structural and functional measures of social relationships and quality of life among older adults: does chronic disease status matter?. Qual Life Res.

[10] Arden-Close E, McGrath N (2017). Health behaviour change interventions for couples: a systematic review. Br J Health Psychol.

[11] Berg CA, Upchurch R (2007). A developmental-contextual model of couples coping with chronic illness across the adult life span. Psychol Bull.

[12] Bandura A (2004). Health promotion by social cognitive means. Health Educ Behav.

[13] Helgeson VS, Jakubiak B, Van MV, Zajdel M (2017). Communal coping and adjustment to chronic illness: theory update and evidence. Pers Soc Psychol Rev.

[14] Vassilev I, Rogers A, Kennedy A, Koetsenruijter J (2014). The influence of social networks on self-management support: a metasynthesis. BMC Public Health.

[15] Martire LM, Schulz R, Helgeson VS, Small BJ, Saghafi EM (2010). Review and meta-analysis of couple-oriented interventions for chronic illness. Ann Behav Med.

[16] Stahl ST, Rodakowski J, Saghafi EM, Park M, Reynolds CF, Dew MA (2016). Systematic review of dyadic and family-oriented interventions for late-life depression. Int J Geriatr Psychiatry.

[17] Martire LM, Lustig AP, Schulz R, Miller GE, Helgeson VS (2004). Is it beneficial to involve a family member? A meta-analysis of psychosocial interventions for chronic illness. Health Psychol.

[18] Voils CI, Coffman CJ, Yancy WS, Weinberger M, Jeffreys AS, Datta S (2013). A randomized controlled trial to evaluate the effectiveness of CouPLES: a spouse-assisted lifestyle change intervention to improve low-density lipoprotein cholesterol. Prev Med.

[19] Gorin AA, Wing RR, Fava JL, Jakicic JM, Jeffery R, West DS (2008). Weight loss treatment influences untreated spouses and the home environment: evidence of a ripple effect. Int J Obes.

[20] Trief PM, Fisher L, Sandberg J, Cibula DA, Dimmock J, Hessler DM (2016). Health and psychosocial outcomes of a telephonic couples behavior change intervention in patients with poorly controlled type 2 diabetes: a randomized clinical trial. Diabetes Care.

[21] Trief PM, Fisher L, Sandberg J, Hessler DM, Cibula DA, Weinstock RS. Two for one? Effects of a couples intervention on partners of persons with type 2 diabetes: a randomized controlled trial. Diabet Med. 2018;36:473–81. 10.1111/dme.13871 PMC6408270 30485516

[22] Wing RR, Marcus MD, Epstein LH, Jawad A (1991). A “family-based” approach to the treatment of obese type II diabetic patients. J Consult Clin Psychol.

[23] Nathan DM, Buse JB, Davidson MB, Ferrannini E, Holman RR, Sherwin R (2009). Medical management of hyperglycemia in type 2 diabetes: a consensus algorithm for the initiation and adjustment of therapy: a consensus statement of the American Diabetes Association and the European Association for the Study of. Diabetes..

[24] Little RR, Rohlfing CL, Sacks DB (2011). Status of hemoglobin A1c measurement and goals for improvement: from chaos to order for improving diabetes care. Clin Chem.

[25] Tang TS, Funnell M, Sinco B, Piatt G, Palmisano G, Spencer MS (2014). Comparative effectiveness of peer leaders and community health workers in diabetes self-management support: results of a randomized controlled trial. Diabetes Care.

[26] Beverly EA, Miller CK, Wray LA (2007). Spousal support and food-related behavior change in middle-aged and older adults living with type 2 diabetes. Health Educ Behav.

[27] Jing D, Zhaoxia G (2018). Application of couple-centered psychological intervention on in nursing Care of Elderly Patients with type 2 diabetes mellitus. Nurs J Chinese Peoples Liberation Army.

[28] Wooldridge JS. A couples-based approach for increasing physical activity among couples with type 2 diabetes. Denver: University of Colorado; 2017.

[29] Brown H, Prescott R (2006). Applied mixed models in medicine 2nd ed 28. Chichester, West Sussex, England: John Wiley & Sons ltd. applied mixed models in medicine 2nd ed 28.

[30] Prestwich A, Conner MT, Lawton RJ, Ward JK, Ayres K, Mceachan RRC (2012). Randomized controlled trial of collaborative implementation intentions targeting working adults’ physical activity. Health Psychol.

[31] Yeling L, Mingzi L, Hua J, Lei L, Yingying L, Jingjing S (2016). Preliminary practice of non-insulin therapy for structured therapy and education curriculum development in patients with type 2 diabetes. Chinese J Diab.

[32] Michie S, Richardson M, Johnston M, Abraham C, Francis J, Hardeman W (2013). The behavior change technique taxonomy (v1) of 93 hierarchically clustered techniques: building an international consensus for the reporting of behavior change interventions. Ann Behav Med.

[33] Morton K, Sutton S, Hardeman W, Troughton J, Yates T, Griffin S (2015). A text-messaging and pedometer program to promote physical activity in people at high risk of type 2 diabetes: the development of the PROPELS follow-on support program. Jmir Mhealth Uhealth.

[34] Patel MS, Asch DA, Rosin R, Small DS, Bellamy SL, Heuer J (2016). Framing financial incentives to increase physical activity among overweight and obese adults: a randomized, controlled trial. Ann Intern Med.

[35] Patel MS, Asch DA, Rosin R, Small DS, Bellamy SL, Eberbach K (2016). Individual versus team-based financial incentives to increase physical activity: a randomized, controlled trial. J Gen Intern Med.

[36] Harris PA, Taylor R, Thielke R, Payne J, Gonzalez N, Conde JG (2009). Research electronic data capture (REDCap)--a metadata-driven methodology and workflow process for providing translational research informatics support. J Biomed Inform.

[37] Harris PA, Taylor R, Minor BL, Elliott V, Fernandez M, O'Neal L (2019). The REDCap consortium: Building an international community of software platform partners. J Biomed Inform.

[38] Ware J, Snoww K, Ma K, Bg G (1993). SF36 Health Survey: Manual and Interpretation Guide.

[39] Ling W, Xiaodan W, Yumei L, Guihong F, Hong Z (2008). The SF_36 scale is used for the reliability and validity of the elderly. Chin J Gerontol.

[40] Toobert DJ, Hampson SE, Glasgow RE (2000). The summary of diabetes self-care activities measure: results from 7 studies and a revised scale. Diabetes Care.

[41] Huan ZW (2014). Validation of the reliability and validity of the Chinese version of diabetes self-management behavior scale. Chin J Nurs.

[42] Svarstad BL, Chewning BA, Sleath BL, Claesson C (1999). The brief medication questionnaire: a tool for screening patient adherence and barriers to adherence. Patient Educ Couns.

[43] Booth MJRqfe, sport. Assessment of physical activity: an international perspective. 2000;71(sup2):114–20. 10.1080/02701367.2000.11082794 25680021

[44] Deng HB, Macfarlane DJ, Thomas GN, Lao XQ, Jiang CQ, Cheng KK (2008). Reliability and validity of the IPAQ-Chinese: the Guangzhou biobank cohort study. Med Sci Sports Exerc.

[45] Wang Limin ZM, Yuchong L, Zhengjing H, Wei D, Zhenping Z (2018). overall plan for monitoring chronic diseases and their risk factors in China in 2013. Chinese J Prev Med.

[46] Rifas-Shiman SL, Willett WC, Lobb R, Kotch J, Dart C, Gillman MW (2001). PrimeScreen, a brief dietary screening tool: reproducibility and comparability with both a longer food frequency questionnaire and biomarkers. Public Health Nutr.

[47] Wan Qiaoqin SS (2009). Reliability and validity of diabetes self-efficacy energy meter. Nurs Res.

[48] Wichit N, Mnatzaganian G, Courtney M, Schulz P, Johnson M (2018). Psychometric testing of the family-Carer diabetes management self-efficacy scale. Health Soc Care Community.

[49] Helgeson VS, Berg CA, Kelly CS, Van Vleet M, Zajdel M, Tracy EL (2019). Patient and partner illness appraisals and health among adults with type 1 diabetes. J Behav Med.

[50] Stephens MAP, Franks MM, Rook KS, Masumi I, Hemphill RC, Salem JK (2013). Spouses’ attempts to regulate day-to-day dietary adherence among patients with type 2 diabetes. Health Psychol.

[51] Sullivan TR, White IR, Salter AB, Ryan P, Lee KJ (2018). Should multiple imputation be the method of choice for handling missing data in randomized trials?. Stat Methods Med Res.

[52] Harden SM, Smith ML, Ory MG, Smith-Ray RL, Estabrooks PA, Glasgow RE (2018). Re-aim in clinical, community, and corporate settings: perspectives, strategies, and recommendations to enhance public health impact. Front Public Health.

[53] Glasgow RE, Estabrooks PE (2018). Pragmatic Applications of RE-AIM for Health Care Initiatives in Community and Clinical Settings. Prev Chronic Dis.

