# Supplementary material for: Couple-based collaborative management model of type 2 diabetes mellitus for community-dwelling older adults in China: protocol for a hybrid type 1 randomized controlled trial
Source: BMC Geriatr. 2020 Mar 30;20:123. doi: 10.1186/s12877-020-01528-5 (PMC7106607; doi:10.1186/s12877-020-01528-5)
Supplement: Supplementary file 2 — Additional file 2. Activity planning demonstration. A demonstration of the intervention implemented on participants specific to each single day of the week. [file 12877_2020_1528_MOESM2_ESM.docx]

**Additional file 2. Activity planning demonstration**

| **Activity planning demonstration of CET** | | | | | | |
| --- | --- | --- | --- | --- | --- | --- |
| **Time** | **Place** | **Method** | **Aim** | **Barriers** | **Solutions** (How does partner assist?) |  |
|  |  |  |  |  |  |  |
| Monday-Wednesday-Friday (after supper) | Park | Warm up for 10 minutes | Moderate aerobic exercise ≥3~5 d/w, daily step count ≥7000 steps | Washing dishes after supper | We will do the dishes together to save time |  |
|  |  | We will walk for 40 mins, |  |  |  |  |
|  |  | Sweating slightly |  |  |  |  |
| Tuesday-Thursday (afternoon) | Home | Warm-up for 10 minutes | Flexibility, balance and muscle-strengthening activities ≥2-3 d/w | Worry about falling;  pick up grandkid from school | Look after each other;  the partner will pick up the kid interchangeably |  |
|  |  | Standing on each leg for 5 minutes |  |  |  |  |
|  |  | Flexibility exercise for 30 minutes |  |  |  |  |
| Saturday morning | Park | Warm up for 10 minutes |  | Rain | Change to indoor activity |  |
|  |  | I do Tai Chi and partner does square dancing, totally 40 minutes |  |  |  |  |
|  |  |  |  |  |  |  |
|  |  |  |  |  |  |  |

*Activity aims reference: exercise 3 times per week, 90 minutes once, include: balance exercise for 15 minutes + suppleness exercise for 15 minutes + aerobic exercise for 30 minutes + strength training for 30 minutes (American diabetes, 2019).
