# Supplementary material for: Couple-based collaborative management model of type 2 diabetes mellitus for community-dwelling older adults in China: protocol for a hybrid type 1 randomized controlled trial
Source: BMC Geriatr. 2020 Mar 30;20:123. doi: 10.1186/s12877-020-01528-5 (PMC7106607; doi:10.1186/s12877-020-01528-5)
Supplement: Supplementary file 3 — Additional file 3. Evaluation of couple intervention. Evalutaion of patients and their spouses during intervention implementation. [file 12877_2020_1528_MOESM3_ESM.docx]

**Additional file 3. Evaluation of couple intervention**

| **Percentage** | **strongly disagree** | **disagree** | **agree** | **strongly agree** |
| --- | --- | --- | --- | --- |
| **Patient evaluation** |  |  |  |  |
| 1. The course enhanced my **knowledge** about diabetes, such as medication, and self-examination. | 1 | 2 | 3 | 4 |
| 1. My **ability** to control my blood glucose has improved since participating in the course. | 1 | 2 | 3 | 4 |
| 1. The course has helped me over my **diabetic management** such as a balanced diet and everyday exercise. | 1 | 2 | 3 | 4 |
| 1. I have paid more attention to **foot care** since participating in the course. | 1 | 2 | 3 | 4 |
| 1. Attending the course with my partner was instructive **to me**. | 1 | 2 | 3 | 4 |
| 1. My partner's **recognition** of diabetes has promoted since participating in the course. | 1 | 2 | 3 | 4 |
| **Spouse evaluation** |  |  |  |  |
| 1. The course enhanced my **knowledge** about diabetes, such as medication, and self-examination. | 1 | 2 | 3 | 4 |
| 1. My **ability** to interact with my partner on diabetes has strengthened since participating in the course. | 1 | 2 | 3 | 4 |
| 1. My **ability** to **allocate responsibility** for diabetes care has improved since participating in the course. | 1 | 2 | 3 | 4 |
| 1. My **ability** to **render assistance** to my partner over diabetic management has improved since participating in the course. | 1 | 2 | 3 | 4 |
| 1. Attending the course with my partner was instructive **to me.** | 1 | 2 | 3 | 4 |
| 1. My partner's **recognition** of diabetes has promoted since participating in the course. | 1 | 2 | 3 | 4 |
